# The female geriatric proximal humeral fracture: protagonist for straight antegrade nailing?

**DOI:** 10.1007/s00402-017-2767-y

**Published:** 2017-07-29

**Authors:** Richard A. Lindtner, Franz S. Kralinger, Sebastian Kapferer, Clemens Hengg, Markus Wambacher, Simon A. Euler

**Affiliations:** 1 Department of Trauma Surgery, Medical University Innsbruck, Anichstr. 35, 6020 Innsbruck, Austria; 2 Department of Trauma Surgery, Wilhelminenspital, Montlearstr. 37, 1160 Vienna, Austria

**Keywords:** Proximal humeral fracture, Straight antegrade nail, Entry point, Proximal anchoring point, Rotator cuff, Osteoporotic fractures

## Abstract

**Introduction:**

Straight antegrade humeral nailing (SAHN) has become a standard technique for the surgical fixation of proximal humeral fractures, which predominantly affect elderly females. The nail’s proximal anchoring point has been demonstrated to be critical to ensure reliable fixation in osteoporotic bone and to prevent iatrogenic damage to the superior rotator cuff bony insertion. Anatomical variations of the proximal humerus, however, may preclude satisfactory anchoring of the nail’s proximal end and may bare the risk of rotator cuff violation, even though the nail is inserted as recommended. The aim of this study was to evaluate the anatomical suitability of proximal humeri of geriatric females aged 75 years and older for SAHN. Specifically, we sought to assess the proportion of humeri not anatomically amenable to SAHN for proximal humeral fracture.

**Materials and Methods:**

A total of 303 proximal humeri of 241 females aged 75 years and older (mean age 84.5 ± 5.0 years; range 75–102 years) were analyzed for this study. Multiplanar two-dimensional reformations (true ap, true lateral, and axial) were reconstructed from shoulder computed tomography (CT) data sets. The straight antegrade nail’s ideal entry point, “critical point” (CP), and critical distance (CD; distance between ideal entry point and CP) were determined. The rate of proximal humeri not anatomically suitable for SAHN (critical type) was assessed regarding proximal reaming diameters of currently available straight antegrade humeral nails.

**Results:**

Overall, 35.6% (108/303) of all proximal humeri were found to be “critical types” (CD <8 mm) as to the recommended minimal proximal reaming diameter of 10 mm of straight antegrade nails currently in use. Moreover, 43.2% (131/303) of the humeri were considered “critical types” with regard to the alternatively used larger proximal reaming diameter of 11.5 mm. Mean CD was 9.0 ± 1.7 mm (range 3.5–13.5 mm) and did not correlate with age (*r* = −0.04, *P* = 0.54). No significant differences in CD and rate of “critical types” were found between left and right humeri as well as between females aged between 75 and 84 years (*n* = 151) and females aged 85 and older (*n* = 152).

**Conclusions:**

More than a third of proximal humeri of geriatric females are “critical types” as to SAHN and may, therefore, be at risk for procedure-related complications, such as rotator cuff violation, fixation failure, and potential malreduction. In view of this finding, we recommend to routinely analyze multiplanar CT reformations of the uninjured contralateral side prior to surgery to improve selection of patients for SAHN and to minimize foreseeable complications. For “critical type” humeri, an alternative surgical procedure should be considered.

## Introduction

The proximal humeral fracture (PHF) is the third most frequent fracture in geriatric patients [1]. Surgical treatment is generally considered for displaced or unstable fracture patterns, depending on the individual elderly patient’s demands and personal needs as well as on the specific medical and social conditions. If treated surgically, straight antegrade humeral nailing (SAHN) offers a variety of potential advantages in this context, compared to alternative fixation techniques: high primary stability and high initial weight bearing capacity, minimal invasive approach, and reduced time of surgery [2–4]. The incidence of osteoporotic PHFs has substantially increased over the last decades [5–9] and beyond that, the percentage of fractures treated surgically considerably raised [6, 8, 10–12]. As elderly females are by far at highest risk to sustain a PHF [13], members of this selective group of patients seem to be protagonists for SAHN.

**Fig. 1:**
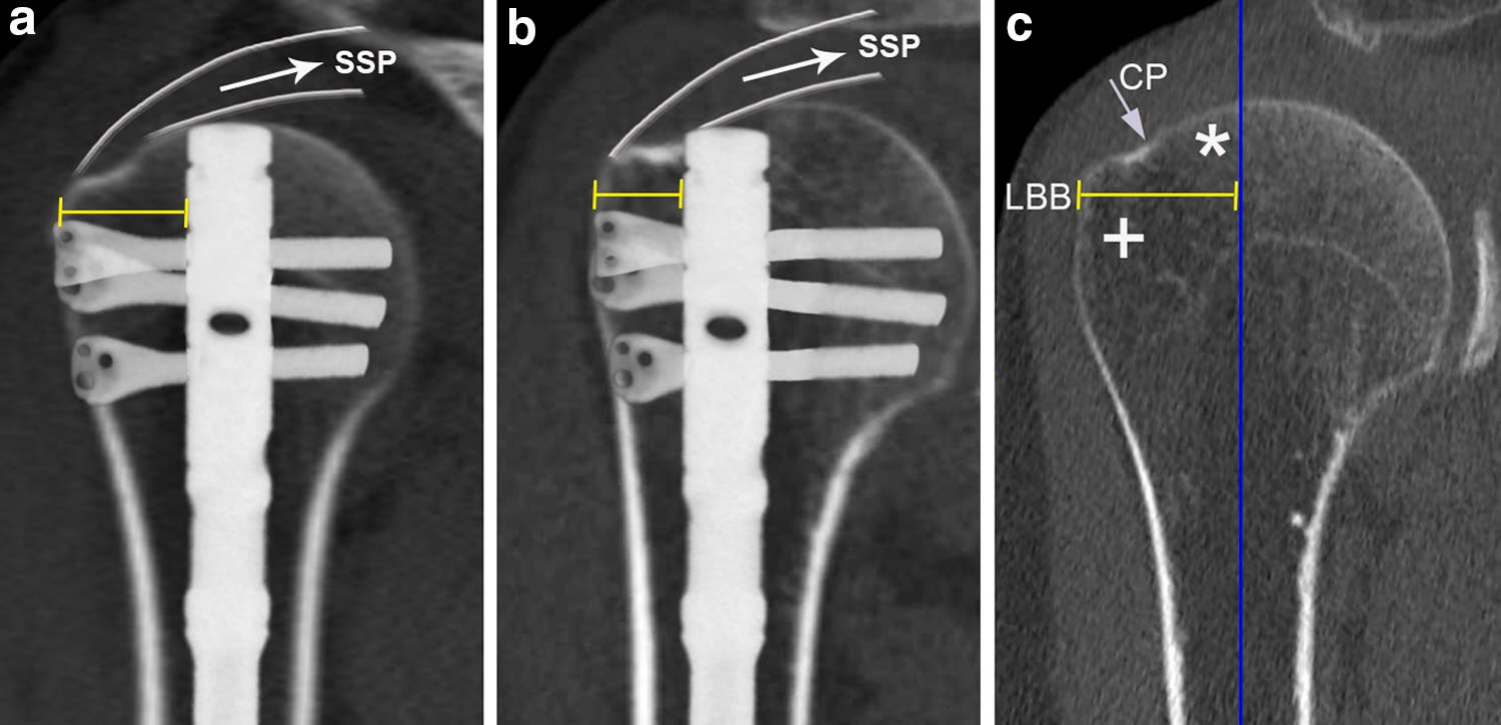
Representative illustration of straight antegrade nail position and actual lateral bony bridge [LBB; referenced to the nail’s lateral rim (*yellow bar*)] in a “safe” (**a**) and a “critical type” (**b**) proximal humerus. Representative illustration of the LBB (referenced to the* vertical axis* through the center of the humeral medullary cavity) (**c**) consisting of the dense subchondral bone medial to the critical point (CP) (*asterisk*) as well as the less dense bone lateral to the CP (*plus symbol*) in the region of the greater tuberosity

When considering SAHN for PHFs, however, the biomechanical principles should be kept in mind. In contrast to curvilinear nails, the straight nail’s entry point is medialized. The medialization of the nail’s entry point preserves a larger amount of bone lateral to the nail’s tip [lateral bony bridge (LBB), Fig. 1] and allows the nail’s proximal end to anchor in the zone of dense subchondral bone to better counteract varus displacing forces. This proximal anchoring point (also known as “fifth anchoring point”) of straight antegrade nails has been regarded to play a critical role to enhance stability of fracture fixation and to resist varus displacing forces. A recent biomechanical study corroborated this hypothesis and clearly showed that the absence of a proximal anchoring point significantly decreases construct stability and results in significantly lower loads to failure [14]. However, variations in proximal humerus anatomy may preclude a proper straight nail’s entry point through the articular surface at the top of the humeral head and, even in SAHN, may result in an undesirably lateral entry point (Fig. 1b). The latter again may not only inevitably lead to rotator cuff violation, but also result in an unfavorable proximal anchoring point and thus decreased stability of osteoporotic fracture fixation.

The aim of this study was to evaluate the anatomical suitability of geriatric proximal humeri for SAHN. We sought to assess the proportion of humeri not amenable to SAHN due to anatomical variations carrying the risk for procedure-related complications, such as rotator cuff violation and compromised stability of osteoporotic fracture fixation. We specifically focused on elderly females aged 75 years and older as these patients are by far at the highest risk for PHF and have been deemed protagonists for SAHN.

## Methods

For this study, the University Department of Radiology Picture Archive and Communications System (PACS) was screened for shoulder computed tomography (CT) scans obtained from female patients aged 75 years and older during routine workup for various reasons, such as fracture exclusion or instability diagnostics (Aquilion Premium CT scanner, Toshiba America Medical Systems, Inc., Tustin, CA, USA). Exclusion criteria were any signs of recent or previous fracture, neoplastic or inflammatory lesion, malformation, and internal hardware. A total of 303 consecutive shoulder CT scans from 241 patients were collected, anonymized, and retrospectively evaluated. In 62 of the 241 patients, bilateral measurements were performed, while in 101 patients only the left and in 78 patients only the right humeri were evaluated, because CT scans of the contralateral humerus were either not performed or ineligible due to exclusion criteria. This resulted in a total of 163 left and 140 right humeri. According to national regulations and institutional guidelines for ethical review, formal institutional review board approval was not required for this anonymized retrospective anatomical study.

**Fig. 2:**
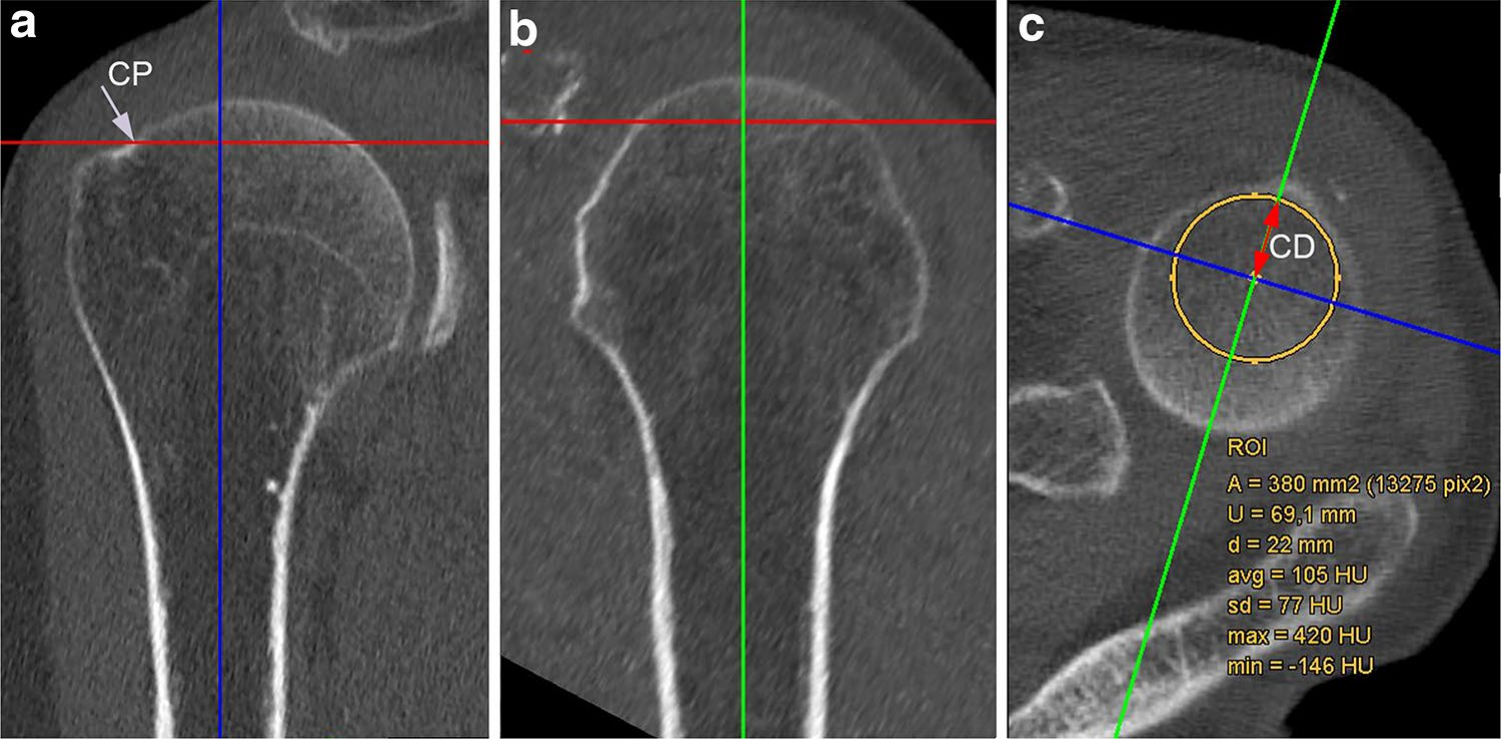
Multiplanar two-dimensional CT reformations of the proximal humerus in three perpendicular planes: true anteroposterior (**a**), true lateral (**b**), and axial (**c**). Critical distance (CD) was defined as the distance between the straight antegrade nail’s ideal entry point (indicated by the* vertical axis* through the center of the humeral medullary cavity extrapolated through the humeral head) and the critical point (CP), and was assessed in the axial reformation at the level of the CP (**c**) (see text for details)

Multiplanar two-dimensional reformations in three perpendicular planes were reconstructed at 0.63 mm from axial CT data sets using the IMPAX EE R20 imaging software (Agfa HealthCare N.V., Mortsel, Belgium). The anatomical suitability of proximal humeri for straight antegrade nailing was evaluated using the anatomical criteria and measurements described by Euler et al. [15] (Fig. 2). In brief, the ideal entry point for a straight antegrade nail at the humeral head was determined by adjusting a vertical axis to the exact center of the proximal humeral shaft and parallel to the anatomical shaft axis in the true anteroposterior as well as true lateral reformation (Fig. 2a, b). The extrapolation of this vertical axis through the humeral head marked the straight antegrade nail’s ideal entry point. The “critical point” (CP) was defined as the transition of the subchondral bone of the humeral head into the cortical bone in the true anteroposterior reformation (Figs. 1c, 2a). The critical distance (CD) was defined as the distance between the straight antegrade nail’s ideal entry point and the CP, and was assessed in the axial reformation at the level of the CP (Fig. 2a, c). For CD measurements, a circular region of interest (ROI) was centered on the vertical axis through the center of the humeral medullary cavity in the axial reformation; the ROI’s radius was manually set to the largest possible extent to make sure to not compromise the CP. Consequently, the ROI’s radius equates to the CD, while the ROI’s diameter equates to the maximum proximal reaming diameter for straight antegrade nailing without violation of the supraspinatus tendon insertion. As the currently available straight antegrade nails’ diameter starts with 9.5 mm (corresponding to a recommended minimal proximal reaming diameter of 10 mm), Euler et al. [15] advised not to go below a CD of 8 mm to avoid violation of the rotator cuff and still allow for proper fixation by ensuring a zone of dense subchondral bone of at least 3 mm lateral to the nail’s tip. Accordingly, all evaluated humeri were divided into “safe types” (CD ≥8 mm) and “critical types” (CD <8 mm) as to the recommended minimal proximal reaming diameter of 10 mm (corresponding to a minimal proximal reaming radius of 5 mm). Moreover, “safe types” (CD ≥8.75 mm) and “critical types” (CD <8.75 mm) were determined with regard to the alternatively used larger proximal reaming diameter of 11.5 mm.

SPSS 16.0 (SPSS Inc., Chicago, IL, USA) was used for statistical analysis. Metric scaled data are reported as arithmetic mean ± standard deviation and categorical data as absolute numbers and percentages. A Student’s *t* test was used for comparisons between left and right humeri as well as for comparisons between age groups (75–84 vs. 85 years and older). Pearson correlation was used to examine the relationship between CD and age. For comparisons between left and right humeri in patients with bilateral measurements, a paired samples *t* test was applied. Fisher’s exact test was used for categorical data. Statistical significance was defined as *P* < 0.05.

## Results

A total of 303 proximal humeri of 241 patients were analyzed for this study. The mean age of all individuals was 84.5 ± 5.0 years (range 75–102 years). Mean CD was 9.0 ± 1.7 mm (range 3.5–13.5 mm) and did not correlate with age (Pearson correlation, *r* = −0.04, *P* = 0.54). Overall, 35.6% (108/303) of all proximal humeri were found to be “critical types” (CD <8 mm) as to the recommended minimal proximal reaming diameter of 10 mm of straight antegrade nails currently in use. Moreover, 43.2% (131/303) of all proximal humeri exhibited a CD <8.75 mm and are considered “critical types” with regard to the alternatively used larger proximal reaming diameter of 11.5 mm.

Comparisons of humeri of patients aged 75–84 years (*n* = 151) and patients aged 85 and older (*n* = 152) did not reveal any significant differences in CD [<85 years: 9.2 ± 1.6 vs. ≥85 years: 8.9 ± 1.8, *P* = 0.26 (Student’s *t* test)] and percentage of “critical types” [CD <8 mm: <85 years: 33.1% vs. ≥85 years: 38.2%, *P* = 0.40 and CD <8.75 mm: <85 years: 42.4% vs. ≥85 years: 44.1%, *P* = 0.82 (Fisher’s exact test for both comparisons)].

Comparisons of the left and right humeri in the 62 individuals with bilateral measurements did not yield any significant differences in CD [left: 8.9 ± 1.8 vs. right: 8.8 ± 1.8, *P* = 0.33 (paired samples *t* test)] and percentage of “critical types” [CD <8 mm: left: 45.2% vs. right: 41.9%, *P* = 0.86, and CD <8.75 mm: left: 51.6% vs. right: 53.2%, *P* = 1 (Fisher’s exact test for both comparisons)]. Similarly, no significant differences in CD and percentage of “critical types” were found when comparing all the left (*n* = 163) and all the right (*n* = 140) humeri [*P* = 0.29, 0.47 (CD <8 mm) and 0.49 (CD <8.75 mm), respectively].

## Discussion

The most important finding of this study was that more than a third of proximal humeri of females aged 75 years and older appeared to be not anatomically suitable for SAHN of PHFs. Specifically, 35.6% (108/303) of all humeri analyzed appeared to be “critical types” as to the minimal proximal reaming diameter of 10 mm recommended for straight antegrade nails currently in use. Moreover, 43.2% (131/303) of proximal humeri were designated “critical types” as to the alternatively used larger proximal reaming diameter of 11.5 mm. However, SAHN of “critical type” humeri may expose these patients to the risk of significant procedure-related complications, such as rotator cuff violation, fixation failure due to a weak proximal anchoring point, and potential malreduction.

Geriatric females are at the highest risk to sustain a PHF and thus have been deemed protagonists for SAHN. The incidence of PHF markedly increases with age and females are at more than 2.5-fold higher risk to sustain a PHF than males [8, 13]. Geriatric females, therefore, obviously represent a key target group for SAHN. Nevertheless, studies assessing the anatomical suitability of proximal humeri of geriatric females for SAHN have been lacking so far.

If surgical treatment is considered for geriatric PHF, SAHN may be an appealing option. Varus failure is known to be the most common failure mode following surgical fixation of PHFs [16–18]. Varus displacing forces typically develop during a fall on the outstretched arm or repetitively whenever the patient is standing up from a chair. In this context, straight antegrade humeral nails appear to provide a variety of biomechanical advantages, as compared to locking plates and curvilinear antegrade humeral nails, primarily owing to their medialized entry point [14]: (1) shorter lever arm, potentially counteracting varus displacing forces superiorly; (2) preservation of the superior rotator cuff footprint; (3) anchorage of the nail’s proximal end in the densest subchondral zone at the apex of the humeral head enhancing construct stability; (4) larger amount of bone lateral to nail’s proximal end (LBB) to resist varus displacing forces; and (5) prevention of inadvertent entry through the fracture zone in case of a fractured greater tuberosity.

**Fig. 3:**
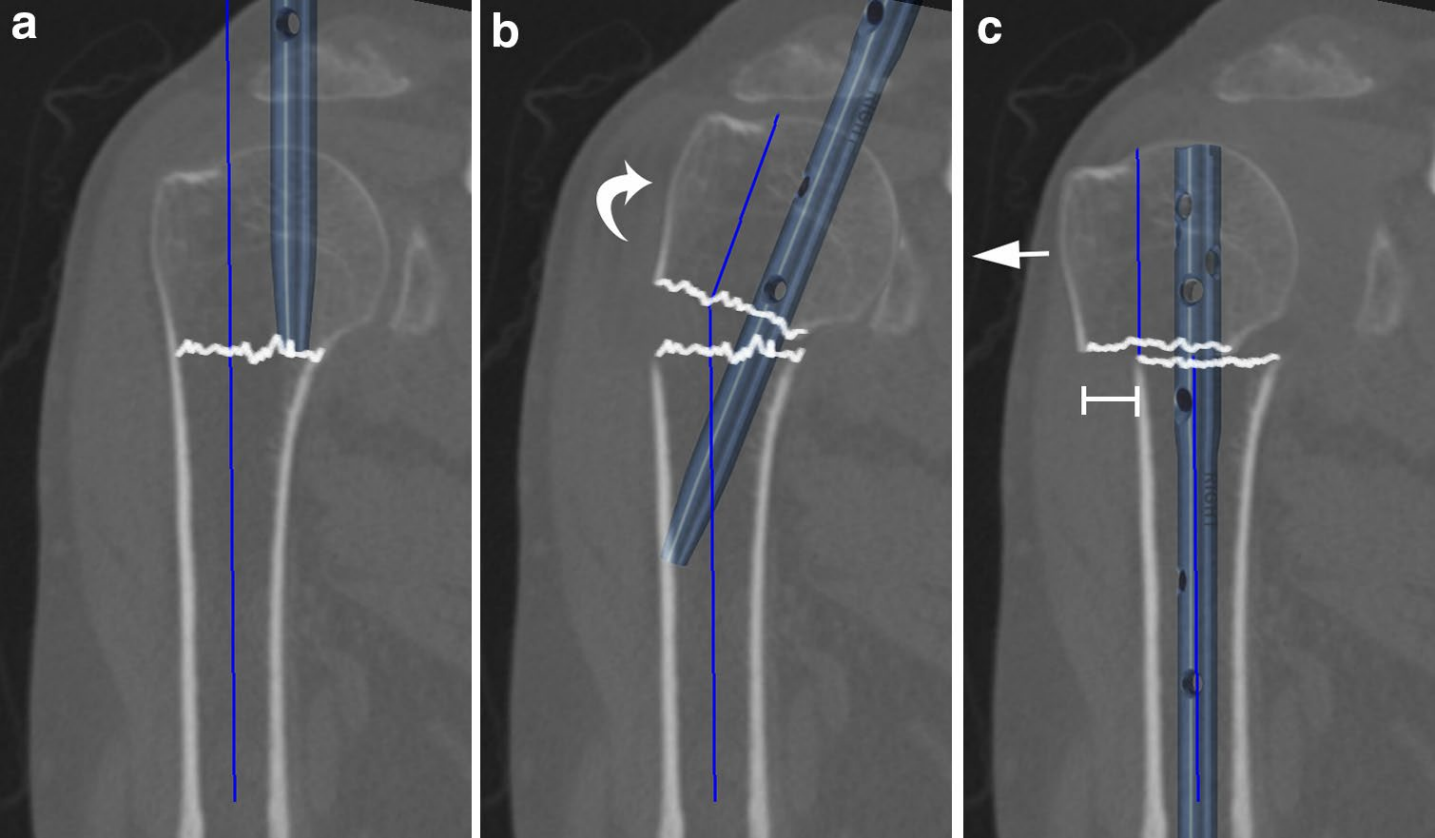
Representative illustration of potential sequelae of using the recommended straight nail’s entry point at the apex of the humeral head for “critical type” proximal humeri. As the medullary canal is typically considerably lateral to the nail’s recommended entry point (**a**), advancing the nail into the humeral medullary cavity requires varus tilt and/or lateral translation of the head fragment (**b**), and nail insertion may result in malreduction and translational displacement of the humeral head fragment to lateral relative to the shaft (**c**)

However, anatomical suitability of the proximal humerus for SAHN is a prerequisite for proper nail insertion, reliable fracture fixation, and avoidance of rotator cuff violation. Conversely, variations in proximal humeral anatomy may result in an unsatisfactory nail position, decreased construct stability, rotator cuff violation, and potential malreduction, even though the nail is inserted as recommended by the manufacturers. The CD is defined as the distance between the straight antegrade nail’s entry point and the CP, and CD minus the nail’s radius consequently equates the extent of subchondral bone lateral to the nail’s lateral rim. The smaller the CD, the greater the risk that (1) the nail does not anchor the dense subchondral bone at the humeral head, (2) the LBB is undesirably small, and (3) the rotator cuff is violated (Fig. 1b). The LBB consists of the bone stock lateral to the nail’s proximal end and thus comprises the portion of dense subchondral bone lateral to the nail’s tip (equivalent to CD minus the nail’s radius) (Fig. 1c, asterisk) as well as the adjacent bone lateral to the CP, i.e., the greater tuberosity (Fig. 1c, plus symbol). Bone mineral density and strength has been reported to be highest in the medio-dorsal and cranial aspect of the humeral head and much lower in the region of the greater tuberosity [19, 20]. Therefore, for two-part fractures, it seems reasonable to assume that the nail’s proximal anchoring point is weakened if the CD is undesirably small, as the dense subchondral bone bridge lateral to the nail’s tip will be reduced or even diminished and thus only the less dense bone at the greater tuberosity may resist varus deforming forces (Fig. 1b, c). In three-part fractures including a greater tuberosity fragment, a sufficient subchondral bone bridge lateral to the nail’s tip is even more important as the greater tuberosity is separated from the humeral head and, therefore, cannot function as a bony resistance to varus displacing forces. An undesirably small CD may not only result in a weakened proximal anchoring point, but also preclude adequate reduction if the recommended nail’s entry point at the apex of the humeral head is chosen. In proximal humeri with a rather small CD, the humeral medullary canal is typically considerably lateral to the nail’s recommended entry point at the top of the humeral head (Fig. 3a). In this situation, advancing the nail into the humeral medullary cavity (after insertion via the top of the humeral head) requires varus tilt and/or lateral translation of the head fragment (Fig. 3b), and nail insertion may result in varus malreduction and/or translational displacement of the humeral head fragment to lateral relative to the shaft (Fig. 3c). Malreduction and an insufficient medial cortical support constitute risk factors for fixation failure and varus collapse [21, 22].

For the aforementioned reasons, it is evident that ensuring the anatomical suitability of the proximal humerus prior to SAHN insertion is important to avoid potential procedure-related complications and to make sure that the patient benefits from the biomechanical advantages, this fixation technique may offer. Nevertheless, the anatomical suitability of proximal humeri for SAHN has not yet been elucidated in a large cohort of elderly females. To the best of our knowledge, there is only one large study [15] specifically assessing the anatomical suitability of proximal humeri for SAHN with special regard to the proximal anchoring point and potential rotator cuff violation. However, the mean patients’ age in this study was 45 years and the findings may not be representative for geriatric females. Nonetheless, the authors reported a similar rate of “critical types” of 38.5% in 200 patients with bilateral measurements using the same anatomical criteria. The percentage of critical type humeri may not be largely influenced by age as also evidenced by our finding that the CD did not correlate with age within our group of patients aged 75 and older. One single study investigated humeri of elderly patients (median age 81 years; range 73–95) and found 42.5% of humeri to be critical types [23]. In contrast to our study, the sample size of this study was rather small and included cadaveric humeri from only ten females. The results of our study furthermore corroborated that the left and right humeri do not significantly differ in CD and percentage of “critical types”, which is well in line with observations from the aforementioned studies [15, 23]. This finding is of clinical relevance as it justifies the use of the uninjured contralateral humerus as a template for preoperative planning and analysis of anatomical suitability for SAHN. Although the incidence of complications has been reported to be markedly lower for straight as compared to curvilinear antegrade nails, it is still substantial, with “symptoms related to rotator cuff” in 34.6% and a re-operation rate of 11.5% [24]. Variations in proximal humerus anatomy might contribute to this high rate of complications, and preoperative analysis of the contralateral humerus may be helpful to improve selection of patients for SAHN and minimize foreseeable procedure-related complications.

This study has inherent limitations. First, it constitutes a descriptive anatomical study using CT data sets and cannot take into account real intraoperative conditions and various degrees of fracture displacement. All measurements were obtained from intact humeri and would presuppose anatomic fracture reduction. Nevertheless, Euler et al. [23] have demonstrated that CT measurements and anatomic measurements of the same cadaveric proximal humeri closely match and that CT measurements allow to reliably predict the entry point for SAHN. Second, we did not conduct repetitive measurements to estimate intra- and interobserver variabilities. However, two previous studies have already shown very low variability and excellent intra-rater and inter-rater reliabilities (intraclass correlation coefficients >0.90) for the measurements used in our study [15, 23].

## Conclusions

More than a third of proximal humeri of geriatric females are “critical types” as to SAHN and may, therefore, be at risk for procedure-related complications, such as rotator cuff violation, fixation failure, and potential malreduction. In view of this finding, we recommend to routinely analyze multiplanar CT reformations of the uninjured contralateral side prior to surgery to improve selection of patients for SAHN and to minimize foreseeable complications. For “critical type” humeri, an alternative surgical procedure should be considered.
